# Potential gains in health expectancy by improving lifestyle: an application for European regions

**DOI:** 10.1186/s12963-018-0181-5

**Published:** 2019-01-17

**Authors:** Koen Füssenich, Wilma J. Nusselder, Stefan K. Lhachimi, Hendriek C. Boshuizen, Talitha F. Feenstra

**Affiliations:** 1 National Institute for Public Health and the Environment, Postbus 1, 3720 BA Bilthoven, The Netherlands; 2 Department of Epidemiology, University Medical Center, Groningen University, Groningen, The Netherlands; 3 Department of Public Health, Erasmus Medical Center, Rotterdam, The Netherlands; 4 Research Group for Evidence Based Public Health, Institute for Public Health and Nursing, University Bremen & Leibniz Institute for Epidemiology and Prevention Research, Bremen, Germany; 5 Department of Agrotechnology and Food Sciences, Wageningen University & Research, Wageningen, The Netherlands

**Keywords:** Life table modeling, Life expectancy in good perceived health, Healthy life years

## Abstract

**Background:**

Prevention aiming at smoking, alcohol consumption, and BMI could potentially bring large gains in life expectancy (LE) and health expectancy measures such as Healthy Life Years (HLY) and Life Expectancy in Good Perceived Health (LEGPH) in the European Union. However, the potential gains might differ by region.

**Methods:**

A Sullivan life table model was applied for 27 European countries to calculate the impact of alternative scenarios of lifestyle behavior on life and health expectancy. Results were then pooled over countries to present the potential gains in HLY and LEGPH for four European regions.

**Results:**

Simulations show that up to 4 years of extra health expectancy can be gained by getting all countries to the healthiest levels of lifestyle observed in EU countries. This is more than the 2 years to be gained in life expectancy. Generally, Eastern Europe has the lowest LE, HLY, and LEGPH. Even though the largest gains in LEPGH and HLY can also be made in Eastern Europe, the gap in LE, HLY, and LEGPH can only in a small part be closed by changing smoking, alcohol consumption, and BMI.

**Conclusion:**

Based on the current data, up to 4 years of good health could be gained by adopting lifestyle as seen in the best-performing countries. Only a part of the lagging health expectancy of Eastern Europe can potentially be solved by improvements in lifestyle involving smoking and BMI. Before it is definitely concluded that lifestyle policy for alcohol use is of relatively little importance compared to smoking or BMI, as our findings suggest, better data should be gathered in all European countries concerning alcohol use and the odds ratios of overconsumption of alcohol.

**Electronic supplementary material:**

The online version of this article (10.1186/s12963-018-0181-5) contains supplementary material, which is available to authorized users.

## Introduction

For quite some time, life expectancy has been the most important measure of population health. However, disease burden has shifted from infectious diseases to chronic diseases, and mortality in many middle- and high-income countries is no longer a sufficient measure of population health. Measures of healthy life expectancy, which focuses on the years spent in good health or without disability [1], are increasingly used.

In the European Union, two health expectancy indicators that are included in the European Core Health Indicators (ECHI) set serve as a basis for monitoring and comparing health status within the EU. These are healthy life years (HLY) and life expectancy in good self-perceived health (LEGPH). HLY is the number of years a person is expected to live without disability, whereas self-perceived health is based on the question, “How good is your health in general?” and hence reflects perception of health rather than actual functioning. Both measures are based on self-report in surveys of the general population [2].

Lifestyle can cause major health losses. In the developed world, unhealthy lifestyle behaviors, such as unhealthy nutritional patterns, too little physical activity, unfavorable BMI, smoking, and alcohol use are among the most important risk factors for health and have been related to large disease burden as measured in disability-adjusted life years (DALYs) [3]. The question might be whether priorities for prevention policy can be derived, considering which target lifestyle factor contributes most to public health in terms of both quality and length of life – that is, as reflected by HLY and LEPGH. More insight into the health losses that can be attributed to the different lifestyle factors supports an answer to such questions. Other important information is how much health gain might be obtained by improvements in lifestyle. Regional variation exists within Europe in the prevalence of different lifestyle risk factors. This translates into regional variation in the health losses attributable to these lifestyle risk factors and in the relative importance of policy aimed at specific risk factors [4, 5].

In the past, studies have mainly been performed to assess the impact of lifestyle on life expectancy, rather than health expectancy, for many European countries [6]. Klijs et al. (2011) [7], compared the impact of BMI, smoking, and alcohol consumption on years lived with disability, but focused only on Dutch data. The GBD study does rank several risk factors by their impact on DALYs and YLLs but does not provide changes in LEGPH and HLY associated with changes in risk factors [3].

Lhachimi et al. compared life expectancy and morbidity-free life years for 11 European countries, but also did not use LEGPH and HLY as outcome measures [8].

Hence it is relevant to analyze the attributable burden of LEPHG and HLY to lifestyle risk factors. The current study focuses on smoking, obesity, and alcohol use and also investigates how much of this burden could be reduced by changes in lifestyle. The problem of answering the latter question is that information has to be obtained to establish a realistic aim for policy. In the current paper, we searched for the best performer within the EU for each lifestyle factor and each age and gender category and considered this as an approximation of such a realistic aim.

In this way, we aim to assess the potential gains in HLY and LEGPH that can be made in 26 EU countries by changing unhealthy lifestyles to the healthiest levels observed within the EU. To keep results insightful, they are presented by aggregates into four regions, namely EU-East, EU-West, EU-South, and EU-North.

This information can help policymakers in making the case for more effective lifestyle-related prevention policy and in setting priorities.

## Methods

To assess the impact of changes in lifestyle on HLY and LEGPH for the European regions, four counterfactual scenarios were created and evaluated with a population health model. The three risk factors considered were unfavorable BMI, smoking, and alcohol consumption.

New odds ratios were estimated for HLY and LEGPH as outcomes, linking the lifestyle risk factor levels to these health outcomes. Additionally, EU-wide input data concerning demography, risk factor epidemiology, and outcomes were gathered. The data were analyzed in a common framework, pooling over countries where possible to increase power and consistency of estimates. Lifestyle scenarios were based on actually observed lifestyle prevalences (healthy example country scenario) as well as a more hypothetical 100% healthy outcome (ideal world scenario).

### Data

Input data consist of risk estimates in the form of odds ratios, baseline values for HLY and LEGPH, demography, and risk factor epidemiology and are presented in more detail in appendix A. Data sources were chosen in 2013 after consultation with an expert panel which commented on a proposal for draft sources. Criteria were accessibility, coverage of the 26 EU countries, consistency among countries, and reliability. While for each single country better sources will exist, no better sources were identified that had equal coverage, accessibility, and consistency. For mortality, we used the relative risks as available from the Dynamo HIA model. These were estimated by dedicated research groups for each risk factor that scrutinized the available European and national data sources (cf www.dynamo-hia.eu, data documentation). HLY and LEGPH are composite outcomes calculated from life expectancy, scores on the General Activity Limitation Indicator (GALI), and on self-perceived health, respectively, based on the EU-SILC survey. Odds ratios for GALI and self-perceived health (SPH) were based on logistic regressions using data from the European Survey of Health and Retirement (SHARE, www.share-project.org), as well as on “L’enquête Handicap-Santé” (HSM, www.insee.fr). Odds ratios were adjusted for age, sex, country, and the level of the other two lifestyle factors and were assumed constant over all countries. The odds ratios for smoking and BMI were stratified by sex and age (50–65 and 65+), since both had a significant influence on the odds ratio. For alcohol, interaction with age was included as a continuous variable, since that model performed best (see Additional file 1).

Data on actual self-reported health outcomes for each country were obtained from the Eurohex website (www.eurohex.eu). The data were interpolated into one-year age groups and smoothed by regression in combination with a smoothing spline, using the R package VGAM for categorical data analysis.

Data on population size by country, age, and sex were obtained from the Eurohex website (www.eurohex.eu) and from the Human Mortality Database (www.mortality.org), estimating the midyear population size of 2010. Data on death counts were obtained from the same two sources.

Data on current prevalence of lifestyle risk factors were obtained from individual-level data from the Eurobarometer survey containing consistent questions on lifestyles across 26 EU countries. For each of the lifestyle variables, we used the most recent Eurobarometer data available when this study was performed in 2013: 2005 for BMI, 2009 for alcohol consumption, and 2012 for smoking (Eurobarometer, https://www.gesis.org/eurobarometer-data-service/home/). A pooled model across all countries was used to estimate risk factor prevalences by country, gender, and age. Models were run for males and females separately, with the risk factor as dependent variable and a country dummy and the interaction between country and age as independent variables. More detailed information about the input data and methodology is available in the report “Comparative efficiency of health systems, corrected for selected lifestyle factors” [9] and in Additional files 1, 2, 3, 4, 5, 6, 7.

### Life table

A Sullivan life table model was applied using the DYNAMO-HIA software and run separately for each country. DYNAMO-HIA (DYNamic MOdeling for Health Impact Assessment) is a population health modeling tool to quantify the health impact of lifestyle changes (www.dynamo-hia.eu [10, 11];). It has already been applied for policy evaluation, e.g., in the areas of alcohol taxation [12], smoking cessation [13], and obesity [14], in 11 EU countries. Hence, the modeling was extended to cover 26 countries.

DYNAMO-HIA both models the evolution of disease in a population over time and constructs period (healthy) life tables. Here we use the latter option, that is, in order to include disability and cover all EU countries, lifestyle risk factors were directly linked to disability, instead of using diseases as an intermediate step. This reduced data requirements. Changes in lifestyle risk factor prevalence were linked to changes in the prevalence of health status by use of odds ratios.

In the model, smoking was categorized into “never smoker,” “ex-smoker,” and “smoker”; BMI was categorized into “BMI lower than 25,” “BMI between 25 and 30,” “BMI above 30”; and alcohol consumption was categorized into four levels based on the amount of grams of alcohol/day, with different boundaries for males and females [15]. By combining these categories for smoking, BMI, and alcohol, a new risk factor was created with 36 categories, reflecting all possible combinations. That is, individuals could be, for instance, non-smoking, overweight, and moderate drinkers. Reliable information on the clustering of lifestyle prevalence for all EU countries was absent, with the different lifestyle estimates only in different survey rounds of the EB data. Hence, it was assumed that smoking, BMI, and alcohol were independent and their clustering was multiplicative, i.e., overweight prevalence was the same among smokers and non-smokers, for instance. This resulted in country-specific age and gender profiles of lifestyle behavior. While a clear simplification, at the aggregate level the impact would be relatively small. Odds ratios were estimated to match this assumption.

### Scenarios

Table 1 lists the scenarios used. The reference or baseline scenario represents countries’ current population and lifestyle prevalence linked to actual mortality and self-rated health and disability. It combines all input data described above in a consistent way, resulting in estimates of country-, age-, and gender-specific HLY and LEGPH.

**Table 1:** Overview of scenarios. Assumptions regarding lifestyle prevalence

Scenario	Short name	Smoking	BMI	Alcohol
0	Reference	As observed	As observed	As observed
1	Best of all/healthy example	Best observed	Best observed	Best observed
2a	Positive all/ideal world all	100% healthy category	100% healthy category	100% healthy category
2b	Positive smoking	100% healthy category	As observed	As observed
2c	Positive BMI	As observed	100% healthy category	As observed
2d	Positive alcohol	As observed	As observed	100% healthy category

The “best of all” scenario applies the most favorable actual lifestyle prevalence of all countries for each age and gender category to all other countries. Thus, this scenario reflects a hypothetical situation, but one based on empirically observed actual lifestyle prevalences. Four further scenarios assumed extremely favorable (all never smokers, no overweight) distributions of risk factors in the populations and serve as a benchmark.

The country-specific outcomes were averaged, using the population size as weights, into four geographical regions as follows [5]:

1. Central-East and Eastern Europe (10 countries): Bulgaria, Czech Republic, Estonia, Hungary, Latvia, Lithuania, Poland, Romania, Slovakia, Slovenia. [Croatia]
2. Nordic countries (3 countries): Denmark, Finland, Sweden. [Iceland], [Norway]
3. Central-West and Western Europe (7 countries): Austria, Belgium, France, Germany, Ireland, Netherlands, UK. [Luxembourg]
4. Southern Europe (6 countries): Cyprus, Greece, Italy, Malta, Spain, Portugal.

Countries in these regions that were not included in the current analysis are in square brackets.

## Results

### Estimation of input data

First, odds ratios and lifestyle prevalences were estimated as input data. For smoking and BMI, odds ratios compared to non-smoking and normal weight were higher for ex-smokers and smokers, and for overweight and obesity. For alcohol, the abstainers have the highest odds ratios, followed by the heaviest drinkers.

As for the lifestyle prevalences, very few people report an alcohol consumption that would place them in the two heaviest drinking categories. Southern and Eastern Europeans abstain from alcohol most often. These regions also show the lowest numbers of people with healthy BMI. Eastern Europeans also smoke the most at older ages. Northern and Western Europeans have the most ex-smokers, while Eastern and Southern European regions have more non-smokers.

### Life table results

**Fig. 1:**
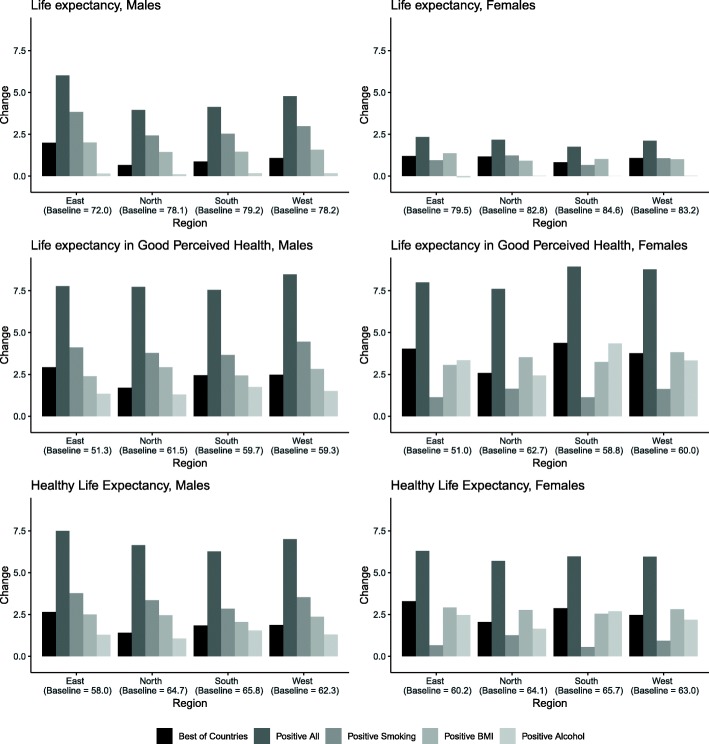
Changes in LE, LEGPH, and HLY

Figure 1 shows the gains in LY, LEGPH, and HLY by improving smoking, BMI, and alcohol, relative to this baseline scenario.

LEGPH was around 60 for Europe except in Eastern Europe, which showed a LEGPH of just above 51.The differences between females and males in LEGPH were much smaller than for LE.

Up to 9 years of extra LEGPH could be gained in the most extremely positive scenario. However, using the more realistic “best of all” scenario, up to 4 years of extra LEGPH could be gained. This gain was obtained for females in Southern Europe.

For HLY, the Eastern European baseline was also lowest; however, this region also had most to gain by improving lifestyle in the “best of all” scenario, 3.3 years in women and 2.6 years in men.

While substantial regional differences could be observed in baseline health outcomes and in total potential gains, the order of importance of the three lifestyles was almost the same for all regions. In the simulations, females had less to gain from reductions in smoking than from improvements in alcohol consumption or a better BMI, whereas males had least to gain from more healthy alcohol consumption and most from a reduction in smoking.

Generally, potential improvements in LEGPH and HLY are larger than those in LE, suggesting a potential for compression of morbidity.

Eastern Europe consistently has lowest outcomes, while Southern and Northern Europe present with best LE, HLY, or LEGPH. Comparing the difference between the best and the worst region for the best of all scenario to those in the baseline scenario, the gap of 5.1 years in LE for women and 7.2 years for men can be reduced by 0.4 and 1.1, respectively, in the best of all scenario. For LEPHG these gaps are larger, 11.6 years for women and 10.1 years for men, and can be reduced by 1.4 or 1.2 years. HLY numbers are more similar to LE, with gaps for women of 5.5 years and for men of 7.7 years, being reduced by 0.4 for women or 0.7 years for men.

## Discussion

In this paper, the differences in current and potential HLY and LEGPH over four European regions were estimated focusing on effects of smoking, alcohol use, and BMI levels.

When using a scenario that assumed lifestyle behavior in all countries was equal to the behavior seen in the country with the healthiest lifestyle, simulations indicate that 1.7 to 2.9 years could be gained in good perceived health for females and 2.6 to 4.4 years for males, with amounts varying by region. For healthy life years, gains were 1.4 to 2.6 years for females and 2.5 to 3.3 years for males.

Eastern Europe has a lower LE, LEGPH, and HLY than the other regions. Part of the gap can be closed by improving the three investigated lifestyles, although a large gap will still remain. However, a reduction of the gap in LE for males of 7.2 to 6.1 seems substantial. The current study suggests that smoking is the leading factor for males, while BMI and alcohol use are most important for females. This order is constant over the different regions, possibly because the same odds ratios were used.

Other studies mostly focused on the relative importance of different risk factors. Klijs et al. [7] found that obesity is the most important factor among the three regarding years with disability in the Netherlands. This is another outcome measure and hence not necessarily at odds with our findings. The Global Burden of Disease study found that for males in Western and Central Europe, smoking is the leading risk factor in terms of DALYs. This is similar to our finding looking at LE, HLY, and LEGPH for males. For females in Western Europe, high BMI and smoking are most important in the Global Buren of Disease study, while we found that both BMI and alcohol have a high impact, so here findings differ a bit.

Our study covered three important and widespread lifestyle-related risk factors that are amenable to change. Other risk factors like physical activity or specific nutritional patterns would be worth investigating but require more and better data than currently available. Furthermore, our current study considers these risk factors to be uncorrelated, while it has been shown that unhealthy behavior often clusters [16]. Even the current choice already suffered from data-related limitations regarding alcohol, as further discussed below. This explains why other studies usually also cover these three factors.

In the Global Burden of Disease study, alcohol use is the second ranking risk factor overall in Eastern Europe, after blood pressure [3]. For our study, a different order was found. However, as discussed below, especially for alcohol consumption, the self-reported character of the data used has its limitations. Contrary to other research, such as the World Health Organization (WHO) status report on alcohol and health, and the Global Burden of Disease study 2013 (GBD), the Eurobarometer data did not suggest higher alcohol use in Eastern Europe than in the other regions. This can potentially be a result of the self-reporting nature of these data. WHO and GBD use triangulation with sales data to correct for this. In our current study, that would have caused inconsistencies with the applied odds ratios, and therefore we did not apply such a correction. While this underreporting might also apply for smoking and BMI, it seems less of a problem when comparing the percentage of participants indicating they are in the high-risk category. Only for alcohol this is remarkably low.

Furthermore, only alcohol quantity is considered, while drinking patterns like binge drinking are ignored. We choose to remain consistent with DYNAMO-HIA in this respect. Additionally, in the current study, we find very strong U-shaped odds ratio curves for alcohol, indicating that not drinking alcohol is worse for disability than drinking. This is potentially due to selection effects, for instance unhealthy heavy drinkers not participating in the survey or abstainers comprising former drinkers who stopped drinking because of ill health. It could also be related to our outcomes, which are in terms of subjective health. Finding a strong U-shape is not uncommon [17] for subjective health outcomes. However, when adjusting for age, sex, years of education, marital status, lack of friends, disability pension, smoking, being an ex-drinker, and reporting a decrease in alcohol intake due to health problems, the U-shape turns into more of a J-shape. Additionally, Stranges et al. [18] show that while current non-drinkers do show lower subjective health, no worse subjective health could be found for lifetime abstainers compared to current drinkers.

At the level of detail provided in the data that are available for all EU countries, such detailed corrections are impossible, implying that the U shape reflected in our odds ratios may overestimate the health benefits of moderate drinking compared to not drinking. However, for mortality, risk follows a J-curve, and this is the more important part of the risk. Therefore, the low impact of alcohol is largely due to the low exposure found in the Eurobarometer. This limitation should be recognized and stresses the need for better alcohol use data.

It should be noted that the odds ratios only include the effects of alcohol on a person’s own health. Increased violence or accidents due to drinking were not included.

With respect to BMI, it should be noted that the “normal” weight group also includes underweight persons, who are likely to have a worse health condition on average. This group, however, is quite small and hence underestimation of the potential health gains of BMI will be limited.

The methods highlight that clustering of risk factors was by assumption multiplicative. That is, interactions more than multiplicative were ignored. This was mainly due to data limitations. At the aggregate level at which our analyses were performed, we consider the influence of this assumption to be limited. DYNAMO-HIA accounts for competing death risks, and we took care not to overestimate the benefits of removing a single risk factor.

A more relevant simplification is that implied by our cross-sectional analyses using the Sullivan method. Such an approach implies the assumption that risk factor distribution, as well as age-specific disability prevalence, is constant over time. In a dynamic situation, risk factor distributions will change, because of cohort effect or behavioral changes. Also, the prevalence of disability is assumed to change directly because of the change in risk factor exposure, while this would occur gradually in a more dynamic simulation. Like any study presenting attributable risks, this should be accounted for when interpreting the results. For instance, smoking levels are currently decreasing for males, while BMI might be increasing. In a future replication of our study we would hence expect to find lower potential effects of smoking reduction and higher potential effects of better BMI. Similarly, the prevalence of female smokers is still increasing in many countries, such that a future study might find higher potential effects for smoking reduction among women.

## Conclusion

In this manuscript, we estimate the gains to be made in life expectancy and health expectancy by improving smoking, BMI, and alcohol consumption levels for four European regions covering almost all European countries. These potential improvements to be made to healthy life years (HLY) and life expectancy in good perceived health (LEGPH) by changing lifestyle had not been modeled previously, while the health expectancy measures HLY and LEGPH play an important role in EU policy.

Especially the modeling of alcohol, based on available data sources, turned out to be quite troublesome. Better data should be gathered in all European countries concerning alcohol use and the odds ratios of overconsumption of alcohol before it is definitely concluded that lifestyle policy for alcohol use is of relatively little importance compared to smoking or BMI.

While the results regarding alcohol should be treated with care, this paper shows what potential gains could be made in the European regions if the best observed levels in the EU were achieved by all countries. Eastern Europe still has a lower LE, LEGPH, and HLY than the other regions, and by improving smoking and BMI, part of this gap can be closed.

## Additional files

Additional file 1: Supplementary description of methods and supplementary tables. (DOCX 327 kb)

Additional file 2: Estimated prevalence of alcohol consumption among males. (JPG 48 kb)

Additional file 3: Estimated prevalence of alcohol consumption among females. (JPG 48 kb)

Additional file 4: Estimated prevalence of tobacco consumption among males. (JPG 47 kb)

Additional file 5: Estimated prevalence of tobacco consumption among females. (JPG 46 kb)

Additional file 6: Estimated BMI distribution of males. (JPG 44 kb)

Additional file 7: Estimated BMI distribution of females. (JPG 45 kb)

## Abbreviations

DYNAMO-HIA: DYNamic Modeling for Health Impact Assessment
GALI: General Activity Limitation Indicator
GBD: Global Burden of Disease study
HLY: Healthy Life Years
LE: Life expectancy
LEGPH: Life Expectancy in Good Perceived Health
SPH: Self-Perceived Health
WHO: World Health Organization
