# Supplementary material for: Potential gains in health expectancy by improving lifestyle: an application for European regions
Source: Popul Health Metr. 2019 Jan 17;17:1. doi: 10.1186/s12963-018-0181-5 (PMC6337827; doi:10.1186/s12963-018-0181-5)
Supplement: Supplementary file 1 — Supplementary description of methods and supplementary tables. (DOCX 327 kb) [file 12963_2018_181_MOESM1_ESM.docx]

**Supplementary data**

More detailed information about the input data and methodology is available in the report “Comparative efficiency of health systems, corrected for selected lifestyle factors” (9). This appendix contains copies of the most relevant information regarding methods from this report. The tables containing the odds ratios and prevalences used in the life table model are shown from page 3 onward below.

**Odds ratios “lifestyle – health outcomes”**

Odds ratios linking risk factor exposure to health outcomes (GALI and self-perceived health) were estimated using the European Survey of Health and Retirement (SHARE, www.share-project.org) for BMI and smoking behavior, and L’enquête Handicap-Santé” (HSM, www.insee.fr) data for drinking behavior. SHARE is an international longitudinal survey that covers a variety of European countries, including both Western and Eastern European countries. SHARE has collected detailed information on lifestyle, health, and health care use of individuals aged 50+ and their spouses. The odds ratios were calculated using wave 4 of the SHARE survey, including the country samples listed in Table 2.5. We used the most recent wave 4, because certain lifestyle questions had changed over time, creating incomparability between the different waves. The HSM is a French survey conducted between March and July 2008. The response rate was 76.6%, corresponding to 29,931 subjects. The questionnaire included questions on GALI limitations and self-perceived health, age, and gender. 14,798 respondents aged 15 years and over also participated in the “auto questionnaire,” which included detailed questions about lifestyle factors, including alcohol consumption, weight, height, and tobacco use.

Odds ratios were estimated using logistic regression models. The two outcome measures, GALI and SPH, were split into two categories. For GALI we distinguished disabled (including both “limited” and “severely limited”) and non-disabled. For SPH, we distinguished “good health” (at least a good perceived health) and “no good perceived health” (remaining categories). Odds ratios were adjusted for age, sex, country, and the other two lifestyle behaviors and were assumed constant over all countries and regions. For alcohol, only French data were used. The odds ratios for smoking behavior and BMI were stratified by sex and age (50-65 and 65+), since both were significant covariates. For alcohol, the models contained age as a continuous variable if the relation to age was significant (p-value < 0.05).

We checked for interactions between the risk factors and age relative to model with only age (either age, or age + age square). For models with age square we checked the interaction with age and age square combined, using the Wald statistic. In case we found a significant interaction, we checked again whether other interactions were needed.
For the age group <15 years, odds ratios were set to 1, and for the age group 15-50 years the odds ratios were assumed to be equal to those of the 50-65 years age group for smoking and overweight. Finally, all resulting odds ratios were checked for consistency and face validity by discussing results with project group members. They have also been presented as part of the interim report to the participants of the expert workshop. For alcohol, the initial SHARE analyses yielded very little contrast in odds ratios, and hence the French dataset was analyzed in addition. The results from this dataset showed more consistency and face validity and were hence preferred over the SHARE results.

The odds ratios for all three lifestyle variables and categories were combined into 36 values representing all possible combinations of risk factor prevalences. To find odds ratios for these combined categories, the single category odds ratios were multiplied with each other.

We are aware that this is a simplifying assumption. However, it suits well to the way the odds ratios have been estimated (correcting for the remaining lifestyle factors). It is also the only way to use the currently available data, with separate sources for each of the lifestyle factors. Only when sufficiently consistent estimates of lifestyle prevalence at the individual level would be available for all countries and for each lifestyle simultaneously, other approaches would be suitable.

Table 1: Smoking and BMI: odds ratios for GALI (limited or severely limited in activities) by gender and age group (95% CI between brackets)

| Sex/age | Male  50-64  (n=11,693) | Male  65+  (n=11,761) | Female  50-64  (n=14,754) | Female  65+  (n=14,696) |
| --- | --- | --- | --- | --- |
| Smoker | 1.80  (1.63–1.99) | 1.28  (1.13–1.45) | 1.14  (1.04–1.25) | 0.92*  (0.80–1.06) |
| Ex-smoker | 1.56  (1.42–1.71) | 1.07  (0.98–1.17) | 1.07  (0.97–1.17) | 1.09  (0.99–1.20) |
| Non-smoker | 1 | 1 | 1 | 1 |
| Obesity | 2.03  (1.82–2.26) | 1.64  (1.46–1.85) | 2.56  (2.32–2.82) | 2.29  (2.07–2.54) |
| Overweight | 1.33  (1.21–1.45) | 1.11  (1.01–1.21) | 1.33  (1.22–1.44) | 1.24  (1.14–1.34) |
| Normal | 1 | 1 | 1 | 1 |

* Set to 1. For females older than 65 years, we found a non-significant odds ratio for smoking of 0.92, implying a protective effect of smoking on disability (GALI). Since this finding is conflicting with literature and it was not significant, we set this value to 1 in the further analyses.

Table 2: Odds ratios for GALI (limited or severely limited in activities) by gender and for selected ages ORs are available as a function of age.

| Sex/age | Male  20 | Male  40 | Male  60 | Male  80 | Female  20 | Female  40 | Female  60 | Female  80 |
| --- | --- | --- | --- | --- | --- | --- | --- | --- |
| High alcohol consumption^1^ | 1.5 | 1.5 | 1.5 | 1.5 | 18.3^*^ | 2.0 | 1.0 | 2.0 |
| Medium alcohol consumption^2^ | 0.9 | 0.9 | 0.9 | 0.9 | 0.4 | 0.7 | 1.1 | 1.3 |
| Minor alcohol consumption^3^ | 1 | 1 | 1 | 1 | 1 | 1 | 1 | 1 |
| No alcohol consumption | 1.7 | 1.7 | 1.7 | 1.7 | 1.5 | 1.5 | 1.6 | 1.6 |

* In the analyses this value was capped at 4.6 to avoid computational difficulties, given the small size of the group and low prevalence of health problems that will only to a minor degree affect the results. 1 Male: >60g/day, Female: >40g/day; 2 Male: 40-60g/day, Female: 20-40g/day; 3 Male: 0.25-40 g/day, Female: 0.25-20 g/day

Table 3: Odds ratios for “Less than good” self-perceived health, by gender and age group (95% CI between brackets)

| Sex/age | Male  50-64  (n=11,692) | Male  65+  (n=11,758) | Female  50-64  (n=14,756) | Female  65+  (n=14,690) |
| --- | --- | --- | --- | --- |
| Smoker | 2.54  (2.29–2.81) | 1.48  (1.30–1.68) | 1.60  (1.45–1.76) | 1.22  (1.06–1.40) |
| Ex-smoker | 1.58  (1.44–1.74) | 1.10  (1.00–1.08) | 1.04  (0.94–1.15) | 1.09  (0.99–1.07) |
| Non-smoker | 1 | 1 | 1 | 1 |
| Obesity | 2.06  (1.85–2.31) | 2.33  (2.07–2.63) | 3.17  (2.86–3.50) | 2.27  (2.05–2.51) |
| Overweight | 1.18  (1.07–1.29) | 1.32  (1.20–1.44) | 1.61  (1.47–1.76) | 1.37  (1.26–1.48) |
| Normal | 1 | 1 | 1 | 1 |

Table 4: Odds ratios for “Less than good” self-perceived health by gender and for selected ages

| Sex/age | Male  20 | Male  40 | Male  60 | Male  80 | Female  20 | Female  40 | Female  60 | Female  80 |
| --- | --- | --- | --- | --- | --- | --- | --- | --- |
| Heavy drinking^1^ | 3.6 | 1.9 | 1.0 | 0.5 | 1.9 | 1.9 | 1.9 | 1.9 |
| Moderate alcohol consumption^2^ | 3.0 | 1.8 | 1.1 | 0.6 | 1.3 | 1.3 | 1.3 | 1.3 |
| Minor alcohol consumption^3^ | 1 | 1 | 1 | 1 | 1 | 1 | 1 | 1 |
| No alcohol consumption | 2.9 | 2.3 | 1.8 | 1.4 | 2.0 | 2.0 | 2.0 | 2.0 |

1 Male: >60g/day, Female: >40g/day; 2 Male: 40-60g/day, Female: 20-40g/day; Male: 0.25-40 g/day, Female: 0.25-20 g/day

## Estimating lifestyle prevalence

Our main data source was the Eurobarometer survey containing consistent questions on lifestyles across EU countries. The Eurobarometer data were available at the individual level. The lifestyle questions were not systematically included in all waves of the Eurobarometer survey. Therefore, reference years differed. For each of the lifestyle variables we used the most recent Eurobarometer data available: 2005 for BMI, 2009 for alcohol consumption, and 2012 for smoking. The Eurobarometer data contained relatively few observations per country, while we needed estimates for each category, age and gender. Hence, data were pooled across all countries using country dummies to obtain prevalence estimates by country, gender, and age using maximal power. In order to predict smoothed estimates, a multinomial “vector generalized additive model” was applied, using the R package VGAM. Several country-specific dummies were included to allow flexibility in the location and shape of the relation between age and lifestyle. Likelihood ratio tests were applied to find the best smoothing parameter. However, these tests revealed a rather high value for smoothing, around 10 or 11. Upon visual inspection, a lower value of 6 was applied in all analyses to avoid too “bumpy” curves.

***Survey questions: BMI***

For overweight, the questions used were obtained from Eurobarometer survey no. 64.3 from November-December 2005 (http://www.gesis.org/).

We used the following questions to calculate BMI:

- Q.D5 How tall are you (in cm) without shoes?

- Q.D6 How much do you weigh (in kg) without shoes and clothes?

BMI was calculated using the basic formula (BMI=weight (in kg)/height (in m²). The following three BMI classes were distinguished:

- BMI<25

- BMI>=25 & BMI<30 (overweight)

- BMI>=30 (obesity)

***Survey questions: tobacco***

For tobacco, the question used were from Eurobarometer survey no. 77.1 from February-March 2012 (http://www.gesis.org/).

The question used was:

- EB72.3 QD1: “Regarding smoking cigarettes, cigars, or a pipe, which of the following applies to you?” (READ OUT – ONE ANSWER ONLY)

The following answer categories were available: You currently smoke (M); You used to smoke but you have stopped; You have never smoked; DK (do not know/no answer). Category DK was left out for the final pooled analysis but is presented in the raw data. It was assumed these were proportionally distributed over the other three categories. Finally, three categories remained:

- never smokers

- former smokers

- current smokers

***Survey questions: alcohol***

For alcohol, we used the questions from the Eurobarometer survey no. 72.3, which was conducted in October 2009 (http://www.gesis.org/).

Questions used were:

- QC1a: During the past 12 months, did you drink any alcoholic beverage (beer, wine, sprits, cider, or other local beverages)?

Answers: Yes; No; DK/Refusal

- QC1c: Did you drink any alcoholic beverages (beer, wine, spirits, cider, or other local beverages) in the last 30 days?

Answer

s: Yes; No; DK/Refusal.

QC2: In the last 30 days, how many times did you drink any alcoholic beverages?

Answers: Daily; 4–5 times a week; 2–3 times a week; Once a week; 2–3 times a month; Once; Don’t remember/Refusal (spontaneous)

- QC3: On a day when you drink alcoholic beverages, how much do you usually drink?

Answers: 1-2 drinks; 3-4 drinks; 5-6 drinks; 7-9 drinks; 10 drinks or more; It depends (Spontaneous); DK/Refusal

These questions were combined to calculate the average daily amounts consumed in grams. The midpoints of the answer categories from QC3 were multiplied with the assumed average 12 grams of alcohol per drink: 1.5*12 g; 3.5*12g; 5.5*12g; 8*12g; 10*12g. These results were then multiplied with the frequencies from QC2 to obtain total weekly and average daily intake. Persons answering yes to either QC1a or QC1c, but having a NA on questions QC2 and/or QC3 were categorized as drinking rarely, that is, 0-0.025 g daily.

Next, four alcohol consumption categories were distinguished, using the cutoff points from WHO (see table below). The two highest WHO categories were combined into a single highest category. Also, the categories lifetime abstainer and former drinker were combined, and people indicating very small annual intake were added to this category of “not to rare drinkers.”

Table 5: Alcohol consumption levels, grams per day

| Men | 0-0.025 | 0.025-40 | 40-60 | 60+ |
| --- | --- | --- | --- | --- |
| Women | 0-0.025 | 0.025-20 | 20-40 | 40+ |

This resulted in three datasets with individual data containing information on country, age, and gender, as well as lifestyle category (either overweight [3 categories], smoking habit [3 categories], or drinking habit [4 categories]).


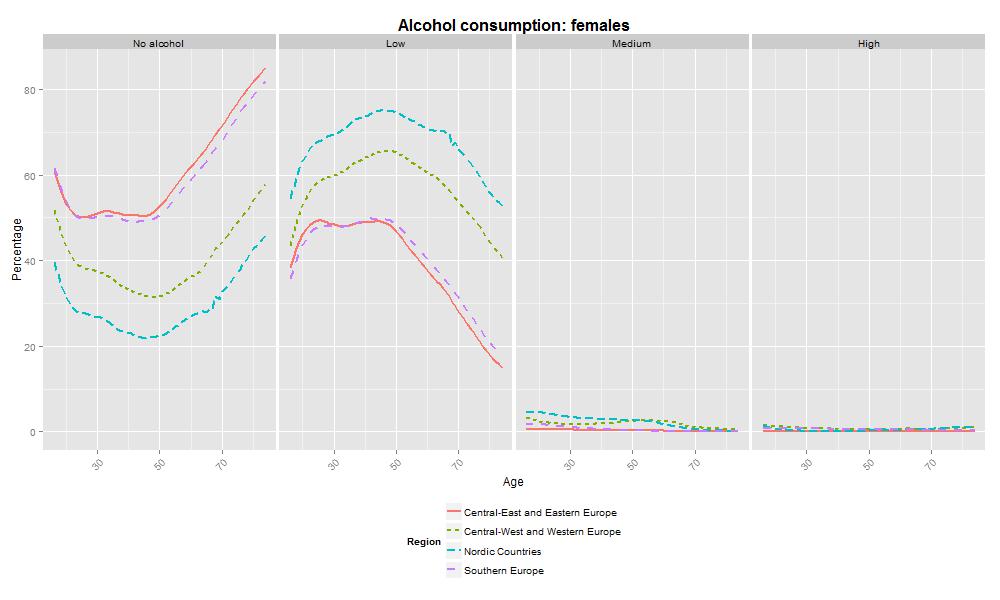

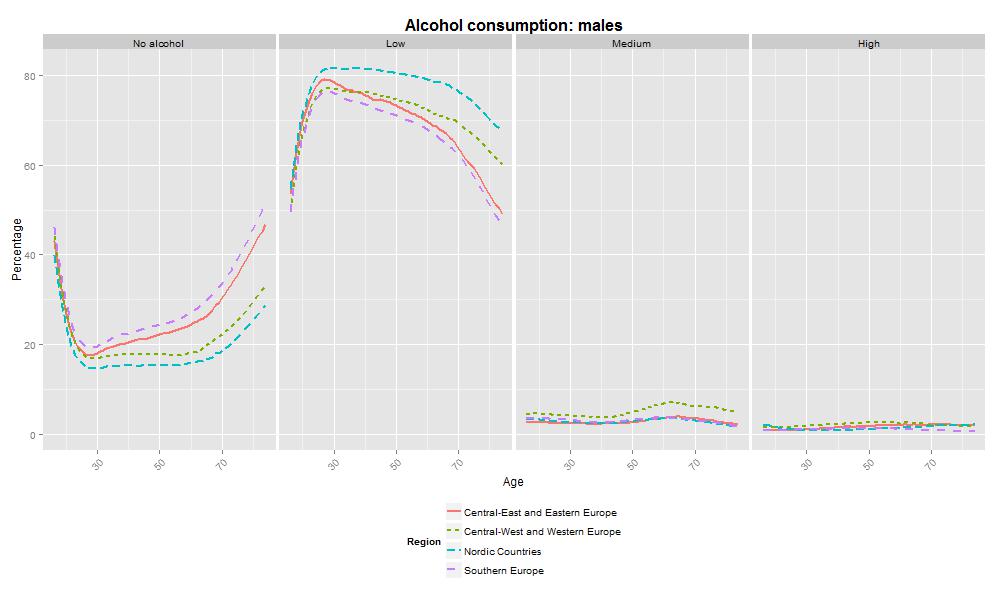

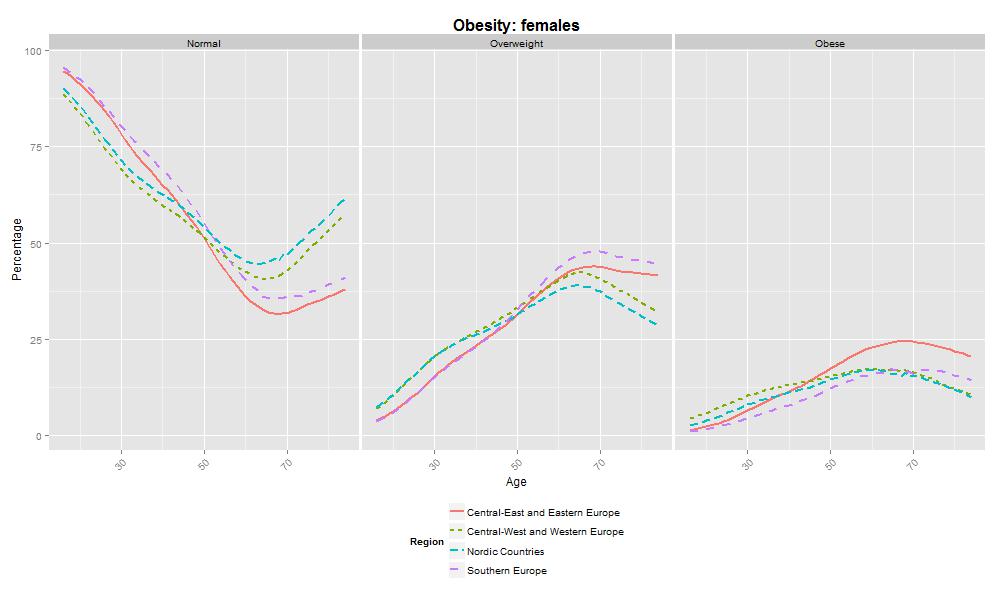

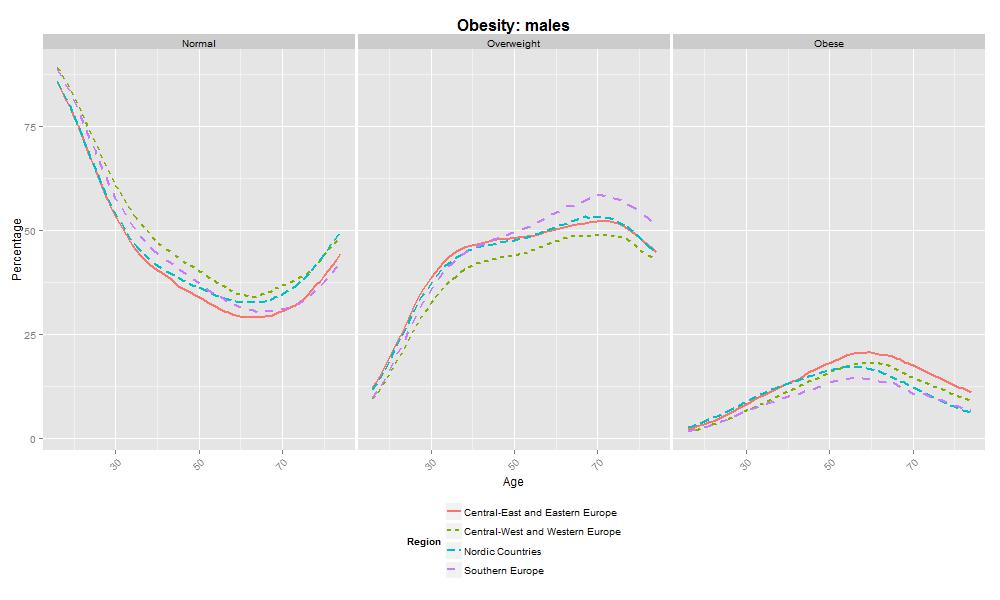

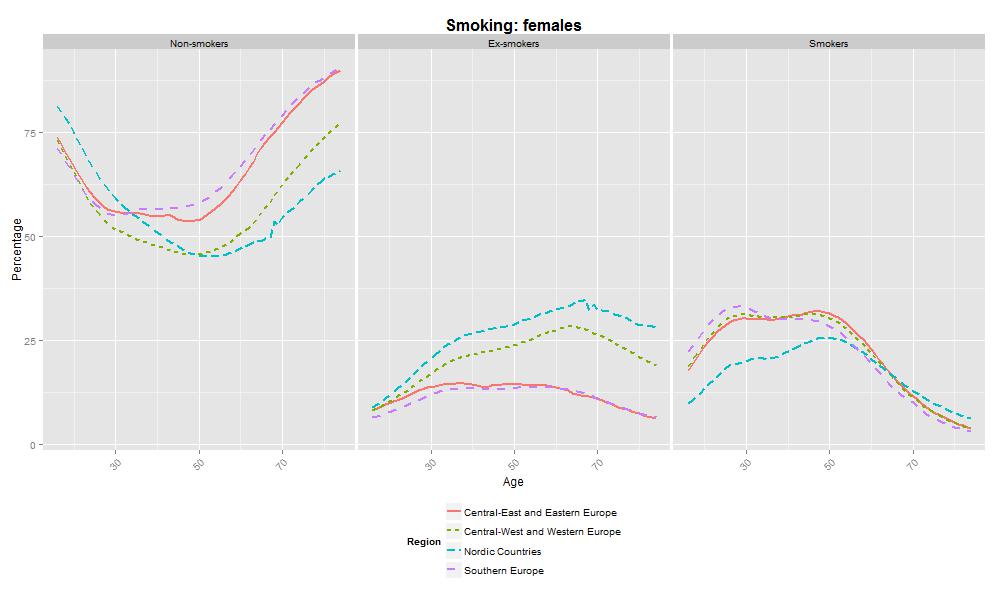

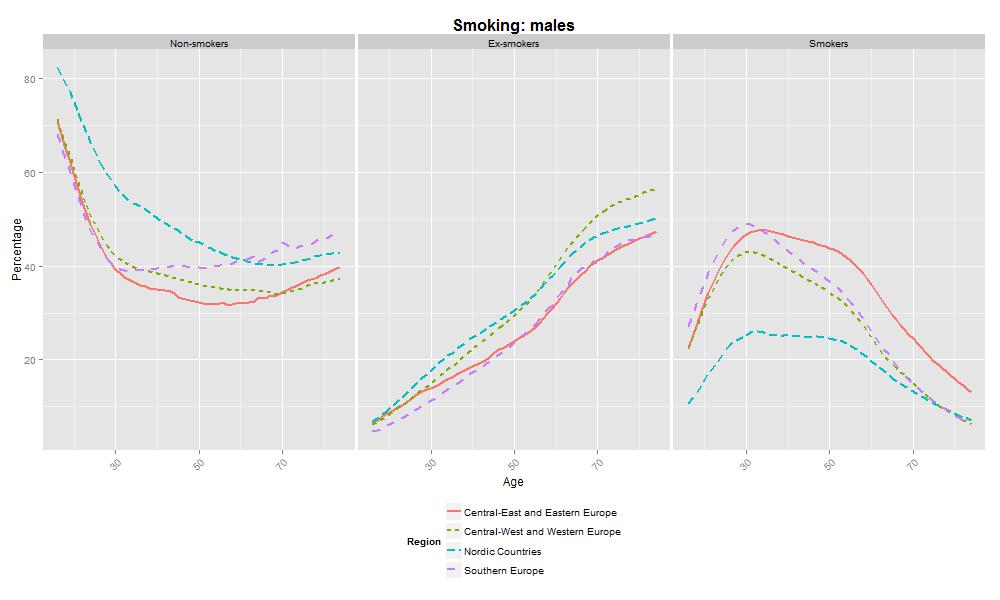


## Applying the Sullivan method using DYNAMO-HIA

The DYNAMO-HIA model produced mortality probabilities, prevalence of self-rated health/disability by age, gender, and country, as well as life expectancy and health expectancy (HLY, LEGHP) in various scenarios in which risk factor levels were varied. Health outcomes at the population level were adjusted using the relative risks (for mortality) and odds ratios (for self-reported health and disability) that were associated with changes in lifestyle.

**Life table model (DYNAMO-HIA)**

We used the DYNAMO-HIA (DYNamic MOdeling for Health Impact Assessment) Model to calculate lifestyle standardized outcomes (www.dynamo-hia.eu; Boshuizen et al. 2012). DYNAMO-HIA is a modeling tool to quantify the health impact of lifestyle changes. The range of countries and many scenarios involved in our study make it more practical and less error-prone to use this dedicated software. DYNAMO-HIA includes checks of the input data (e.g., total prevalence of risk factors equals 100%) and has data storage capacities. The model was extended to allow for multiple risk factors so that in the scenario analyses all three risk factors could be changed simultaneously.

We ran DYNAMO-HIA with lifestyle risk factors directly linked to general health outcomes in terms of mortality (relative risks) and disability (odds ratios). This avoids the use of less comparable and often unavailable country-specific disease prevalence, incidence, and mortality data, as well as disease- and country-specific cost data that will be impossible to obtain for all countries included.

The baseline scenario reflects the current situation, that is, it represents countries’ current lifestyle prevalence in relation to current health outcomes in terms of mortality and quality of life. Using odds ratios and relative risks, total mortality and quality of life were attributed to in total 36 different lifestyle categories, specific for age and gender.

**Population**

Data on population size by country, age, and sex was obtained from the Eurohex website (www.eurohex.eu). In case the Eurohex population data were incomplete, data from the Human Mortality Database were added. In addition, for Romania the highest age group was 85+ years. For Romania, we estimated the age distribution of the 85+ population using the distribution of similar countries with regard to geographical location and life expectancy, i.e., Hungary (Romania).

We averaged the population estimates of 2010 and 2011 for the midyear population size of 2010. For countries with a population size lower than 2,000,000 (Cyprus, Malta, Estonia, and Slovenia) the years 2009, 2010, 2011, and 2012 were averaged.

**Mortality**

Data on death counts were obtained from the Eurohex website, complemented with data from the Human Mortality Database (www.mortality.org) where necessary. Mortality by age and sex was calculated by dividing the death counts by midyear population size of 2010. For the four small countries, the death counts of 2010 and 2011 were averaged and divided by the average population sizes of 2009, 2010, 2011, and 2012.

**Health status**

For GALI, both “limited” and “severely limited” people were considered disabled, and for self-perceived health we distinguished the groups “good health” (at least a good perceived health) and “no good perceived health” (remaining categories). The Eurohex website published these data in 5-year age categories (from 16-19 years to 85+ years). The data were interpolated and smoothed by regression in combination with a smoothing spline, using the R package VGAM for categorical data analysis. The prevalence for 0 to 19 and 85 to 95 were set constant at their 19- and 85-year values.

**Scenarios used in the standardization**

Health outcomes were standardized using different scenarios. These scenarios describe hypothetical changes in the prevalence of lifestyle factors causing changes in health outcomes and health spending. In each of the scenarios, the prevalence of a certain lifestyle variable (e.g., BMI) is set to a certain value for each country, gender, and age stratum. We used the following scenarios:

Scenario “reference”

In the reference scenario, lifestyle prevalences were the smoothed observed data.

Scenario “best of all”

In the “best of all” scenario, we apply the prevalence of the best-performing country to all other countries. A separate adjustment is made for each age group and gender. In other words, in each age-gender stratum a different country may be the best-performing country. “Best-performing” in this case is equal to the highest prevalence in the most favorable category from a health perspective, i.e., the lowest prevalence of current smokers, the highest prevalence of moderate drinkers, and the highest prevalence of no overweight. By using a different reference category in each age-gender stratum, we assume that health behavior and health policy can vary within countries.

For scenario “positive all,” we set the prevalence of the most favorable category to 100% for all three lifestyle variables. In the other three variants, we applied the most favorable category of one of the three lifestyle variables to the entire population and used the observed prevalence for the other two lifestyle variables. This means that in the scenario “Positive alcohol,” we set all the prevalence of moderate drinking to 100% and used the observed prevalence for each BMI category and smoking category. In scenario “Positive BMI,” we set BMI<25 to 100% for all countries and used the observed prevalence for each smoking category and each alcohol use category. In scenario “Positive Smoking,” we set the prevalence of never-smokers to 100% and used the observed prevalence for alcohol use and BMI. These scenarios are called positive alcohol, positive weight, and positive smoking, respectively.

The country specific outcomes were averaged using the population size as weights, into four geographical regions, as follows([4](#_ENREF_4)):

1. Central-East and Eastern Europe (10 countries): Bulgaria, Czech Republic, Estonia, Hungary, Latvia, Lithuania, Poland, Romania, Slovakia, Slovenia. [Croatia]
2. Nordic Countries (3 countries): Denmark, Finland, Sweden. [Iceland], [Norway]
3. Central-West and Western Europe (7 countries): Austria, Belgium, France, Germany, Ireland, Netherlands, UK. [Luxembourg]
4. Southern Europe (6 countries): Cyprus, Greece, Italy, Malta, Spain, Portugal.

Countries in these regions that were not included in the current analysis are in square brackets. Luxembourg was excluded since it is quite small and we suspected a substantial amount of cross-border health care taking place in Germany, France, and Belgium. Croatia was excluded since we did not have access to sufficient data on this country due to its very recent entrance to the EU. Norway and Iceland are not part of the EU.

Table 6: Life expectancy compared to the baseline scenario, by region and gender

|  |  |  | Difference between scenario and baseline scenario | | | | | |
| --- | --- | --- | --- | --- | --- | --- | --- | --- |
| Country | Gender | Baseline  scenario | | Best of all countries | All positive^1^ | Smoking positive^1^ | BMI positive^1^ | Alcohol positive^1^ |
| Central-East and Eastern Europe | Females | 79.5 | 1.2 | | 2.3 | 1.0 | 1.4 | -0.1 |
| Nordic countries | Females | 82.9 | 1.2 | | 2.2 | 1.2 | 0.9 | 0.0 |
| Southern Europe | Females | 84.6 | 0.8 | | 1.8 | 0.7 | 1.0 | 0.0 |
| Central-West and Western Europe | Females | 83.2 | 1.1 | | 2.1 | 1.1 | 1.0 | 0.0 |
| Largest difference* |  | 5.1 | 4.7 | | 4.6 | 4.8 | 4.7 | 5.2 |
| Central-East and Eastern Europe | Males | 72.0 | 2.0 | | 6.0 | 3.8 | 2.0 | 0.2 |
| Nordic countries | Males | 78.2 | 0.7 | | 4.0 | 2.4 | 1.4 | 0.1 |
| Southern Europe | Males | 79.2 | 0.9 | | 4.1 | 2.5 | 1.5 | 0.2 |
| Central-West and Western Europe | Males | 78.2 | 1.1 | | 4.8 | 3.0 | 1.6 | 0.2 |
| Largest difference* |  | 7.2 | 6.1 | | 5.3 | 5.9 | 6.7 | 7.2 |

*1: All positive: 100% in healthiest category for all lifestyle variables; Smoking positive: 100% in healthiest category for smoking; BMI positive: 100% in healthiest category for BMI; Alcohol positive: 100% in healthiest category for alcohol consumption (minor alcohol consumption). *The difference in life expectancy between the lowest and the highest regional value.*

Table 7: Life expectancy in good perceived health (LEGPH) at birth compared to the baseline scenario, by region and gender

|  |  |  | Difference between scenario and baseline scenario | | | | |
| --- | --- | --- | --- | --- | --- | --- | --- |
| Country | Gender | Baseline  scenario | Best of all countries | All positive^1^ | Smoking positive^1^ | BMI positive^1^ | Alcohol positive^1^ |
| Central-East and Eastern Europe | Females | 51.1 | 4.0 | 8.0 | 1.1 | 3.1 | 3.4 |
| Nordic countries | Females | 62.7 | 2.6 | 7.6 | 1.6 | 3.5 | 2.4 |
| Southern Europe | Females | 58.8 | 4.4 | 8.9 | 1.1 | 3.2 | 4.4 |
| Central-West and Western Europe | Females | 60.0 | 3.8 | 8.8 | 1.6 | 3.8 | 3.3 |
| Largest difference* |  | 11.6 | 10.2 | 11.2 | 12.1 | 12 | 10.6 |
| Central-East and Eastern Europe | Males | 51.4 | 2.9 | 7.8 | 4.1 | 2.4 | 1.3 |
| Nordic countries | Males | 61.5 | 1.7 | 7.7 | 3.8 | 2.9 | 1.3 |
| Southern Europe | Males | 59.7 | 2.5 | 7.5 | 3.7 | 2.4 | 1.8 |
| Central-West and Western Europe | Males | 59.3 | 2.5 | 8.5 | 4.5 | 2.8 | 1.5 |
| Largest difference* |  | 10.1 | 8.9 | 10 | 9.8 | 10.6 | 10.1 |

*1: All positive: 100% in healthiest category for all lifestyle variables; Smoking positive: 100% in healthiest category for smoking; BMI positive: 100% in healthiest category for BMI; Alcohol positive: 100% in healthiest category for alcohol consumption (minor alcohol consumption). *The difference in life expectancy between the lowest and the highest regional value.*

Table 8: Healthy life years (HLY) at birth compared to the baseline scenario, by region and gender

|  |  |  | Difference between scenario and baseline scenario | | | | |
| --- | --- | --- | --- | --- | --- | --- | --- |
| Country | Gender | Baseline  scenario | Best of all countries | All positive^1^ | Smoking positive^1^ | BMI positive^1^ | Alcohol positive^1^ |
| Central-East and Eastern Europe | Females | 60.2 | 3.3 | 6.3 | 0.7 | 2.9 | 2.5 |
| Nordic countries | Females | 64.1 | 2.0 | 5.7 | 1.3 | 2.8 | 1.7 |
| Southern Europe | Females | 65.7 | 2.9 | 6.0 | 0.6 | 2.5 | 2.7 |
| Central-West and Western Europe | Females | 62.9 | 2.5 | 6.0 | 0.9 | 2.8 | 2.2 |
| Largest difference* |  | 5.5 | 5.1 | 5.2 | 5.4 | 5.1 | 5.7 |
| Central-East and Eastern Europe | Males | 58.1 | 2.6 | 7.5 | 3.8 | 2.5 | 1.3 |
| Nordic countries | Males | 64.7 | 1.4 | 6.6 | 3.4 | 2.5 | 1.1 |
| Southern Europe | Males | 65.8 | 1.9 | 6.3 | 2.9 | 2.1 | 1.5 |
| Central-West and Western Europe | Males | 62.3 | 1.9 | 7.0 | 3.5 | 2.4 | 1.3 |
| Largest difference* |  | 7.7 | 7 | 6.5 | 6.8 | 7.3 | 7.9 |

*1: All positive: 100% in healthiest category for all lifestyle variables; Smoking positive: 100% in healthiest category for smoking; BMI positive: 100% in healthiest category for BMI; Alcohol positive: 100% in healthiest category for alcohol consumption (minor alcohol consumption). *The difference in life expectancy between the lowest and the highest regional value.*
