# Supplementary figures and images for: Potential gains in health expectancy by improving lifestyle: an application for European regions
Source: Popul Health Metr. 2019 Jan 17;17:1. doi: 10.1186/s12963-018-0181-5 (PMC6337827; doi:10.1186/s12963-018-0181-5)

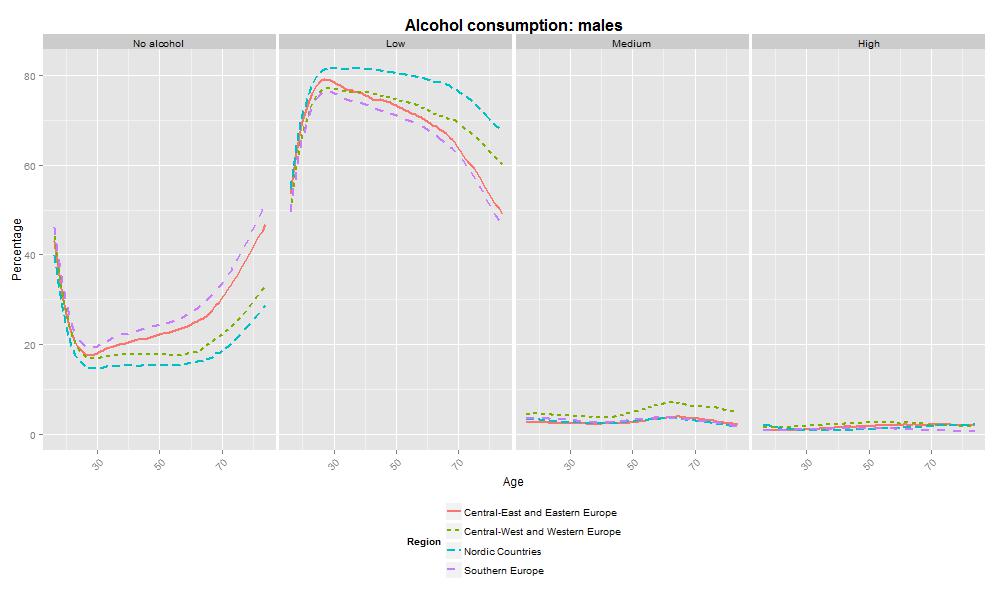

Supplement: Supplementary file 2 — Estimated prevalence of alcohol consumption among males. (JPG 48 kb) [file 12963_2018_181_MOESM2_ESM.jpg]

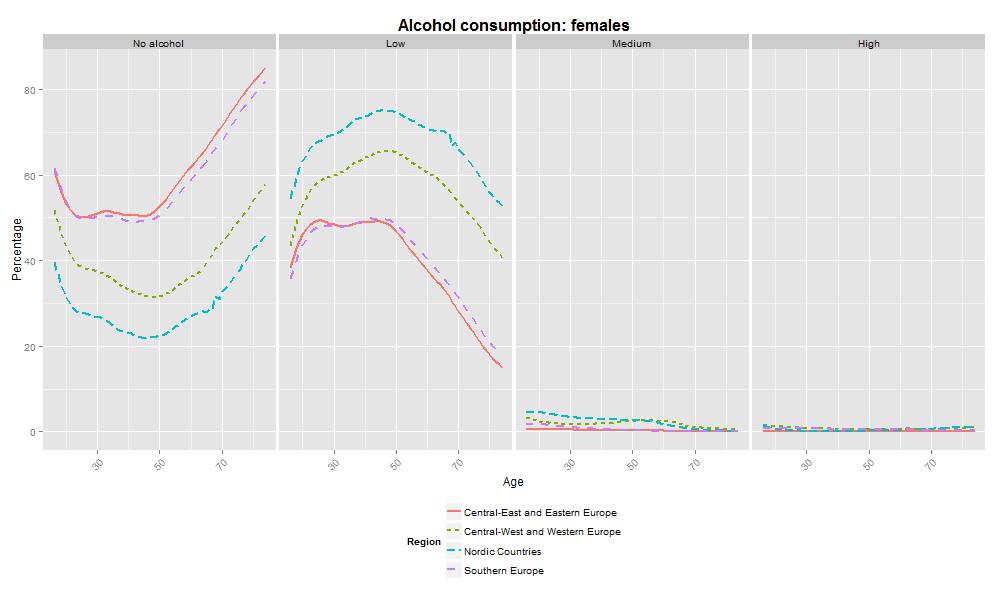

Supplement: Supplementary file 3 — Estimated prevalence of alcohol consumption among females. (JPG 48 kb) [file 12963_2018_181_MOESM3_ESM.jpg]

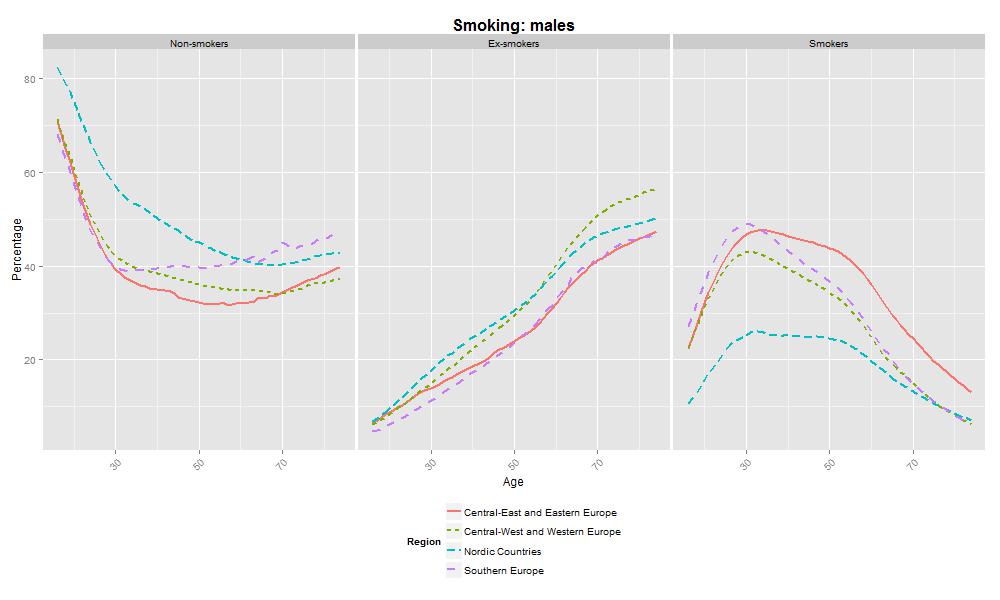

Supplement: Supplementary file 4 — Estimated prevalence of tobacco consumption among males. (JPG 47 kb) [file 12963_2018_181_MOESM4_ESM.jpg]

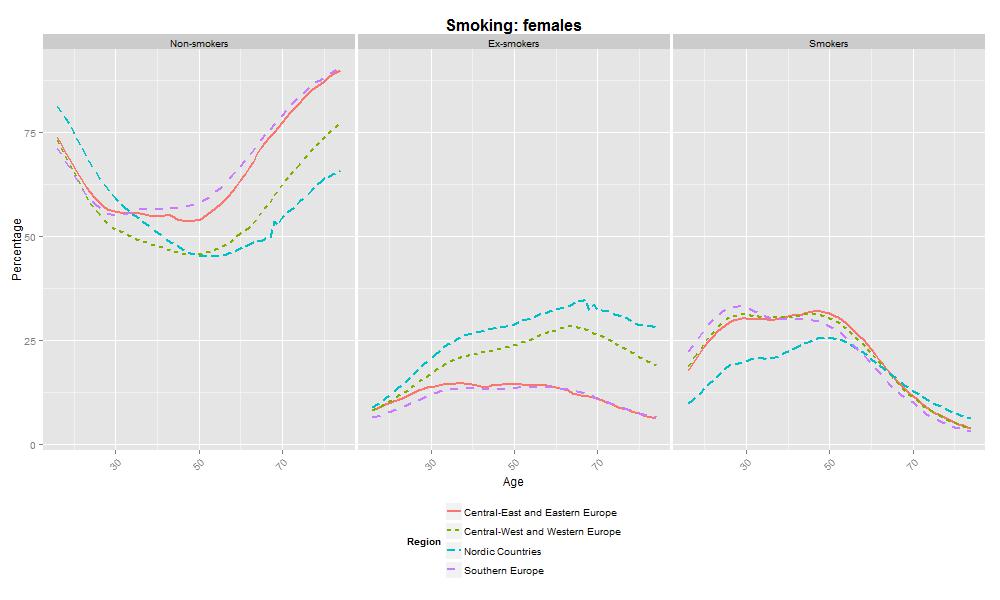

Supplement: Supplementary file 5 — Estimated prevalence of tobacco consumption among females. (JPG 46 kb) [file 12963_2018_181_MOESM5_ESM.jpg]

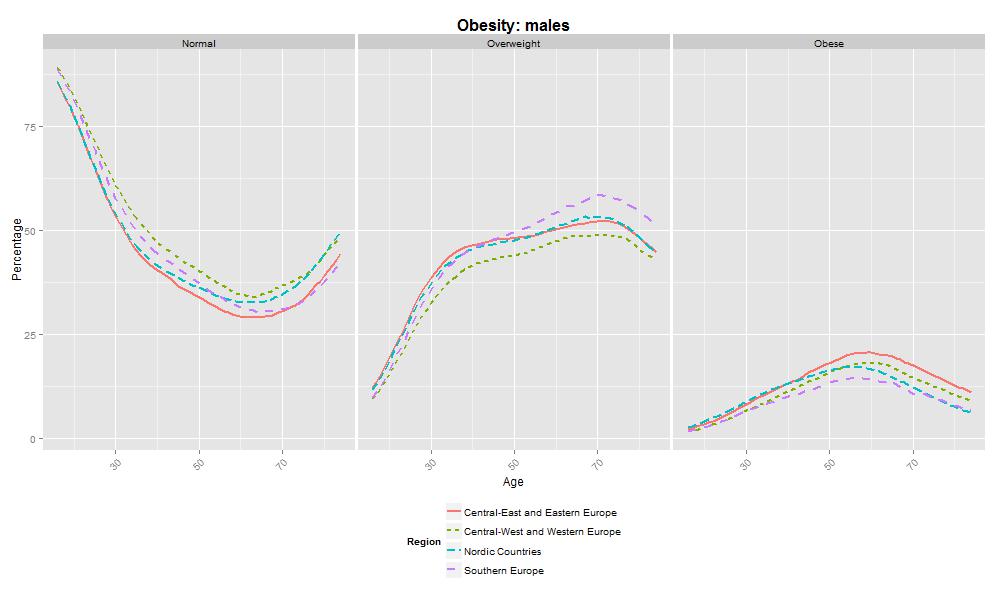

Supplement: Supplementary file 6 — Estimated BMI distribution of males. (JPG 44 kb) [file 12963_2018_181_MOESM6_ESM.jpg]

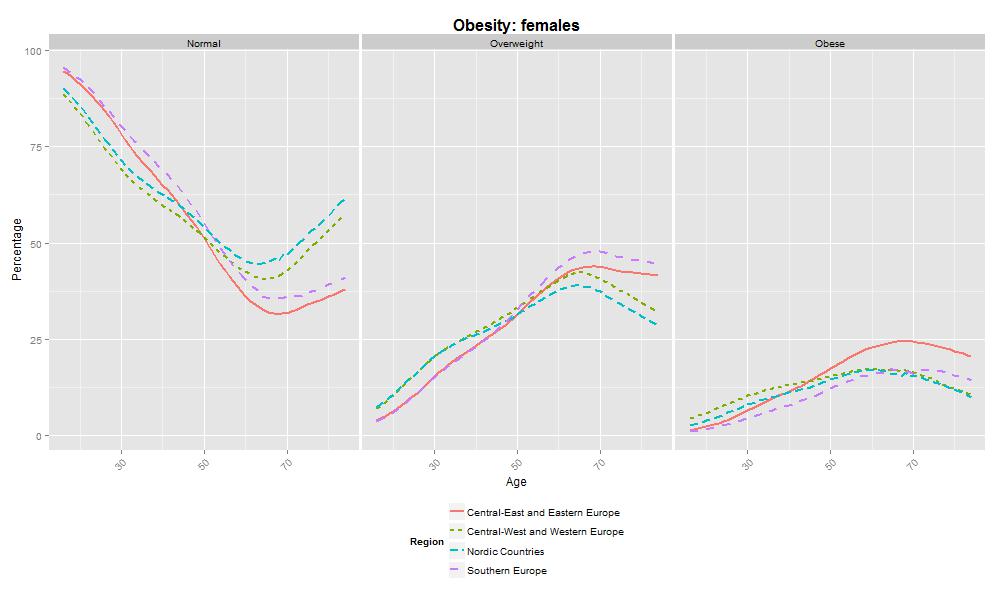

Supplement: Supplementary file 7 — Estimated BMI distribution of females. (JPG 45 kb) [file 12963_2018_181_MOESM7_ESM.jpg]
